# Physiological and metabolic responses to prolonged hypoxia and extreme cold: Preliminary data from the White Mars Antarctica winter expedition

**DOI:** 10.1186/2046-7648-4-S1-A121

**Published:** 2015-09-14

**Authors:** Katie A O'Brien, Ross Pollock, Mike Stroud, Alex Kumar, Robert J Lambert, David A Green, Lindsay M Edwards, Stephen Harridge

**Affiliations:** 1 Centre of Human and Aerospace Physiological Sciences, King's College London, London, UK; 2 NIHR Biomedical Research Centre for Nutrition, Southampton, UK; 3 University Hospitals of Leicester, UK & University of Fribourg, Switzerland; 4 Dept. of Trauma and Orthopaedic Surgery, Royal Infirmary of Edinburgh, UK

## Introduction

The Antarctic winter is amongst the most extreme environments on earth. Human adaptation to this environment, where severe cold is coupled with moderate altitudes, is poorly understood. In this study, a number of physiological and metabolic measurements were made on a small group of trekkers before and after an attempted winter crossing of Antarctica (White Mars Expedition).

## Methods

5 male subjects aged 28-54 yrs were assessed prior to and following a 24 week stay in Antarctica, including 14 weeks above 2,500 m. Measurements included assessment of body fat and bone mineral density (DXA), cardiorespiratory responses to an incremental exercise test, lung and cardiovascular function as well as metabolomic analysis of serum using ^1^H-NMR spectroscopy.

## Results

**Figure 1:**
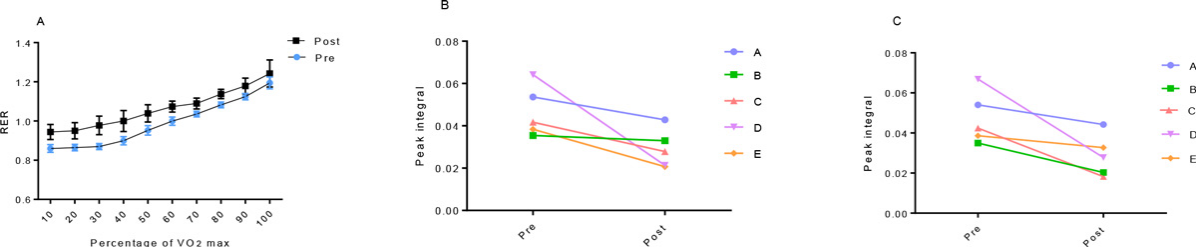
**A, Mean (SD) RER recorded for each percentile of VO_2max _test, changes in the peak integrals of glucose (B) and the fatty acid CH_2 _resonance (C) pre and post expedition**.

Significant changes were found in the following parameters pre to post expedition, identified using a paired Student t test (mean (SD), p < 0.05). There was an increase in % lean tissue (79+4 vs. 81+3%), a decrease in % fat tissue (21(4) vs. 19(3) %) and body fat mass (16(5) vs. 14(4) kg), although whole body weight did not change. Both spine bone mineral density (1.2(0.05) vs. 1.13(0.04) g.cm^2^) and FEV_1_:FVC (68(10) vs. 62(8)) were decreased. VO_2max _did not significantly change from the pre-expedition 42mL.kg.min, however an increase was observed pre to post expedition in the respiratory exchange ratio (RER) at each stage (10%) of the VO_2max _test (Figure 1A). Metabolomics analysis of serum samples revealed changes in two peaks within principal component 2: glucose and a fatty acid CH_2 _resonance (Figure 1B and 1C).

## Discussion

These results are suggestive of a number of physiological changes resulting from prolonged exposure to the Antarctic winter. In particular, we observed a change in metabolic signature involving changes to both glucose and fatty acid homeostasis with a shift towards increased reliance on carbohydrate metabolism during exercise.

## Conclusion

This study has highlighted areas of interest for future investigations into the physiological responses to this unique environment.

